# The association of subjective quality of life with emotion dysregulation and binge-eating pathology in acne vulgaris: a cross-sectional study^؆^

**DOI:** 10.1016/j.abd.2026.501487

**Published:** 2026-11-07

**Authors:** Cagdas Boyvadoglu, Cagla Boyvadoglu, Ayse Serap Karadag

**Affiliations:** a Dermatology Clinic, Adana City Training and Research Hospital, Adana, Türkiye; b Department of Psychiatry, Çukurova University Balcalı Hospital, Adana, Türkiye; c Dermatology Clinic, Private Clinic, Ataşehir, İstanbul, Türkiye

**Keywords:** Acne vulgaris, Binge-eating disorder, Cross-sectional studies, Emotional regulation, Feeding and eating disorders, Quality of life

## Abstract

**Background:**

Acne vulgaris (AV) imposes psychosocial burden beyond lesion severity, yet the specific psychological profiles it produces remain incompletely characterized.

**Objective:**

To examine emotion dysregulation (DER) and binge-eating disorder (BED) psychopathology in AV patients, and evaluate whether subjective quality of life (QoL) mediates the association between objective severity and psychological outcomes.

**Methods:**

This cross-sectional study enrolled 100 AV patients and 105 matched controls. Objective severity (GAGS), QoL (AQLS), DER (DERS-16), and eating psychopathology (EDE-Q-13/BED subscale) were assessed. Bootstrapped mediation analyses and multivariate regression were performed.

**Results:**

AV patients demonstrated significantly higher DERS-16 (*d* = 1.82) and BED subscale scores (p = 0.005) than controls. Group membership predicted DERS-16 and EDE-Q-13-BED scores independently of sociodemographic covariates. GAGS severity showed no direct association with DERS-16 or EDE-Q-13. Crucially, AQLS significantly mediated the effect of GAGS on both DERS-16 (β = 2.747, 95% CI [0.164, 5.837]) and EDE-Q-13 (β = 0.336, 95% CI [0.036, 0.836]); these indirect effects remained robust after adjustment for education, occupation, and income.

**Study limitations:**

Cross-sectional design limits causal inference. Significant sociodemographic differences existed between groups (controlled via regression). Statistical power for GAGS subgroup comparisons was limited. No structured psychiatric interviews were conducted.

**Conclusions:**

Subjective QoL impairment, rather than objective lesion burden, is associated with significant indirect effects on both emotion dysregulation and affect-driven eating pathology in AV patients. Routine dermatological care should integrate QoL assessment and psychological screening, irrespective of clinical severity.

## Introduction

Acne vulgaris (AV) is among the most prevalent chronic inflammatory dermatoses worldwide, affecting approximately 85% of adolescents and persisting into adulthood in a substantial proportion of patients.^1^ Beyond its cutaneous manifestations, AV frequently imposes a psychosocial burden that appears disproportionate to objective lesion burden ‒ a dissociation with direct implications for clinical assessment and management.^2^ While associations between AV and depression and anxiety are reasonably well established, the specific psychological mechanisms and psychiatric profiles that AV may engender in dermatological patients remain insufficiently characterized.

Difficulties in Emotion Regulation (DER) ‒ deficits in the cognitive and behavioral processes by which emotional responses are modulated ‒ have been proposed as a transdiagnostic vulnerability factor in psychodermatology.^3^ In AV specifically, DER has been identified as a potentially stronger predictor of impaired quality of life (QoL) than clinical lesion burden, suggesting that subjective disease perception may be more closely associated with psychological outcomes than objective severity.^3^ This has direct relevance for dermatologists: the absence of severe lesions does not preclude significant psychological morbidity.

A clinically relevant, yet underexplored, behavioral consequence of sustained emotion dysregulation is its potential to promote disordered eating. Maladaptive emotion regulation strategies are robustly associated with a broad spectrum of eating disorder psychopathology across diagnostic categories.^4^ Binge-eating behavior, in particular, may function as a short-term affective coping mechanism, providing transient relief from negative affect ‒ and thereby becoming a self-reinforcing, though ultimately destructive, behavioral strategy. This potential pathway ‒ from the psychological distress of a stigmatizing skin disease, through progressive emotion dysregulation, to compensatory binge eating ‒ may represent a clinically under-recognized risk trajectory in AV patients. Crucially, this profile may be qualitatively distinct from the restraint-based eating disorders driven primarily by sociocultural thin-ideal internalization.

Prior investigations of disordered eating in AV patients have yielded inconsistent results, partly attributable to the use of broad screening instruments without adequate control for sociodemographic confounders that independently predispose to restraint-based eating pathology.^5^, ^6^, ^7^ Furthermore, no prior study appears to have formally evaluated whether objective acne severity has a direct association with psychological outcomes, or whether this relationship is instead explained by the patient’s subjective experience of QoL impairment ‒ a mechanistic question with direct consequences for clinical prioritization.

The present study was designed to address these clinical gaps. We hypothesized that: (i) AV patients would demonstrate significantly elevated DER and BED psychopathology relative to healthy controls, independent of sociodemographic confounders; (ii) these findings would not be directly associated with objective acne severity (GAGS); and (iii) subjective QoL impairment (AQLS) would show a significant mediating role in the association between objective severity and psychological outcomes.

## Methods

### Study design and participants

This cross-sectional study was conducted at the Dermatology outpatient clinic of Adana City Training and Research Hospital. Data collection was conducted between January 2025 and October 2025. The study enrolled 100 consecutive patients aged ≥ 12 years with a clinician-confirmed diagnosis of AV (patient group), and 105 healthy volunteers aged ≥12 years without any known dermatological or psychiatric comorbidities (control group). Control group participants were recruited from individuals who volunteered to take part in the study and were attending the dermatology outpatient clinic in a non-patient capacity, comprising companions and relatives of patients presenting to the clinic, medical intern students rotating through the department, and hospital administrative and ancillary staff. To ensure comparability on key demographic variables, healthy controls were individually matched to the patient group by age and sex distribution.

Exclusion criteria for the patient group included any known psychiatric disorder or current use of psychotropic medication, determined via self-report. This psychiatric exclusion criterion was applied equally to both the patient and control groups.

The study was conducted in accordance with the principles of the Declaration of Helsinki and received ethical approval from the Scientific Research Ethics Committee of Adana City Training and Research Hospital. Written informed consent was obtained from parents or legal guardians, alongside written assent from the participants themselves for underage participants (< 18 years), prior to enrolment, in accordance with current ethical guidelines.

### Clinical assessment

Objective acne severity was assessed in the patient group using the Global Acne Grading System (GAGS), a validated instrument grading lesion severity across six facial and truncal regions, yielding total scores categorized as mild (1–18), moderate (19–30), severe (31–38), and very severe (≥ 39).^8^ Acne-specific QoL was evaluated using the validated Turkish version of the Acne Quality of Life Scale (AQLS), in which higher scores indicate greater QoL impairment.^9^

### Psychometric instruments

All participants completed the validated Turkish version of the Difficulties in Emotion Regulation Scale-Brief Form (DERS-16), a 16-item self-report instrument derived from the original 36-item DERS and designed to assess clinically meaningful dimensions of emotion dysregulation with reduced respondent burden while maintaining strong psychometric fidelity.^10^ The DERS-16 evaluates six conceptually distinct dimensions: (i) non-acceptance of negative emotional responses (the tendency to experience secondary negative reactions to one’s own distress); (ii) difficulties engaging in goal-directed behavior during emotional arousal; (iii) impulse control difficulties in the context of negative affect; (iv) limited access to effective emotion regulation strategies when distressed; (v) lack of emotional awareness; and (vi) lack of emotional clarity regarding one’s affective states. Each item is rated on a five-point Likert scale (1 = almost never to 5 = almost always), with total scores ranging from 16 to 80; higher scores reflect greater overall difficulty in modulating emotional experiences. The scale has demonstrated strong internal consistency, convergent validity, and sensitivity to clinically relevant levels of dysregulation across a range of psychiatric and psychosomatic conditions.^10^ In psychodermatological contexts specifically, elevated DERS scores have been associated with greater QoL impairment and maladaptive coping in patients with chronic inflammatory skin diseases, including AV.^3^, ^5^

Eating disorder psychopathology was assessed using the validated Turkish version of the Eating Disorder Examination Questionnaire short form (EDE-Q-13), a 13-item adaptation of the original 28-item EDE-Q retaining its core psychometric structure with substantially reduced respondent burden.^11^ The EDE-Q-13 evaluates three broad cognitive and behavioral domains central to eating disorder psychopathology: dietary restraint (the intentional and cognitively motivated restriction of food intake); shape concern (overvaluation of body shape and its influence on self-evaluation); and weight concern (preoccupation with body weight and associated distress). Items are scored on a seven-point scale (0–6) reflecting frequency or severity over the preceding 28 days, with higher total scores indicating greater global eating psychopathology. As an instrument whose total score is particularly sensitive to the cognitive restraint and shape- and weight-related preoccupation that characterize restraint-driven eating disorder phenotypes, the EDE-Q-13 total score is substantially influenced by sociocultural factors ‒ including thin-ideal internalization and body dissatisfaction ‒ that vary systematically with socioeconomic and educational status.^11^

Critically, the EDE-Q-13 includes a dedicated Binge-Eating Disorder subscale (EDE-Q-13-BED), which has been validated as a psychometrically distinct factor embedded within the broader instrument yet measuring a qualitatively separate construct.^11^ Unlike the total EDE-Q-13 score ‒ which predominantly reflects cognitive dietary restraint and shape and weight concerns ‒ the EDE-Q-13-BED subscale specifically targets loss-of-control eating episodes and affect-driven binge-eating behavior: that is, recurrent episodes of consuming objectively large amounts of food accompanied by a subjective sense of inability to stop, characteristically precipitated or maintained by negative affective states rather than by cognitive preoccupation with weight or shape. This distinction is of central clinical and methodological relevance to the present study. Restraint-based eating pathology is primarily driven by thin-ideal internalization and the cognitive motivation to restrict dietary intake, and is therefore sensitive to sociocultural and socioeconomic influences. Binge-eating pathology, by contrast, is robustly associated with negative affect, emotion dysregulation, and the use of eating behavior as a maladaptive short-term strategy for affective relief.^4^ Employing both the total EDE-Q-13 score and the EDE-Q-13-BED subscale as separate outcome variables thus allows the present study to distinguish between these two qualitatively distinct eating pathology phenotypes ‒ a methodological granularity absent from prior investigations of disordered eating in AV patients.

### Statistical analysis

Analyses were performed using IBM SPSS Statistics 26.0 and JASP 0.19.3. Descriptive statistics were reported as frequencies and percentages for categorical variables, and as means ± standard deviations for continuous variables. Between-group comparisons of continuous variables were performed using independent-samples *t*-tests; categorical variables were compared using chi-square (χ^2^) tests. One-way ANOVA with post-hoc LSD correction was applied for multi-group comparisons within the patient group. Pearson correlation coefficients were computed to examine associations among continuous variables. Effect sizes were reported as Cohen’s *d* for *t*-tests and partial eta-squared (η²p) for ANOVA.

To evaluate the independence of group-level differences in DERS-16, EDE-Q-13, and EDE-Q-13-BED scores from sociodemographic confounders (educational attainment, occupational status, household income, age, and BMI), three multivariate linear regression models were constructed with group membership as the primary independent variable.

To evaluate the potential mediating role of subjective QoL impairment (AQLS) in the relationship between objective acne severity (GAGS) and psychological outcomes (DERS-16 and EDE-Q-13), two bootstrapped mediation analyses were conducted within the AV patient group (n = 100), using the Hayes PROCESS framework (Model 4) with 5,000 bootstrap resamples.^12^ Statistical significance of indirect effects was determined by 95% bias-corrected bootstrap confidence intervals (CIs) excluding zero. Both mediation models were adjusted for the three sociodemographic variables that showed statistically significant between-group differences ‒ educational attainment, occupational status, and household income ‒ to control for the potential confounding effects of these socioeconomic variables within the patient sample. Post-hoc statistical power analyses were conducted for ANOVA comparisons across GAGS severity subgroups. All tests were two-tailed, with a significance threshold of p < 0.05.

## Results

### Demographic and clinical characteristics

The study enrolled 100 AV patients and 105 healthy controls. Groups were comparable in sex distribution (80.0% vs. 78.1% female; χ^2^(1) = 0.112, p = 0.436), mean age (19.84 ± 5.17 vs. 19.93 ± 4.99 years; p = 0.895), and BMI (22.53 ± 4.10 vs. 22.80 ± 3.36 kg/m^2^; p = 0.606). However, significant between-group differences were identified for educational attainment (χ^2^(4) = 44.893, p < 0.001), occupational status (χ^2^(2) = 38.335, p < 0.001), and income level (χ^2^(4) = 68.181, p < 0.001), with the control group characterized by substantially higher socioeconomic status. These differences were systematically addressed in multivariate regression analyses. Participant characteristics are presented in Table 1, Table 2.

**Table 1:** Demographic and clinical characteristics of study participants.

Variable	Group	Category	n	%	χ^2^ / t	df / SD	p
Gender	Patient	Female	80	80.0	0.112	1	0.436
	Patient	Male	20	20.0			
	Control	Female	82	78.1			
	Control	Male	23	21.9			
Age (years)	Patient	19.84 ± 5.17			−0.132^a^	203	0.895
	Control	19.93 ± 4.99					
BMI (kg/m²)	Patient	22.53 ± 4.10			−0.517^a^	203	0.606
	Control	22.80 ± 3.36					
Disease Duration (years)	Patient	3.69 ± 3.94			−	‒	‒
Prior Treatment		None	43	43.0			
		Topical	20	20.0			
		Systemic antibiotic	18	18.0			
		Isotretinoin	19	19.0			
GAGS Severity		Mild	20	20.0			
		Moderate	54	54.0			
		Severe	26	26.0			

BMI, Body Mass Index; GAGS, Global Acne Grading System.

a *t*-statistic.

**Table 2:** Socioeconomic characteristics of study participants.

Variable	Group	Category	n	%	χ^2^	df	p
Educational Status	Patient	Illiterate	2	2.0	44.893	4	<0.001
	Patient	Primary School	8	8.0			
	Patient	Secondary School	15	15.0			
	Patient	High School	51	51.0			
	Patient	Higher Education	24	24.0			
	Control	Illiterate	0	0.0			
	Control	Primary School	12	11.4			
	Control	Secondary School	8	7.6			
	Control	High School	16	15.2			
	Control	Higher Education	69	65.7			
Profession	Patient	Student	46	46.0	38.335	2	<0.001
	Patient	Employed	24	24.0			
	Patient	Unemployed	30	30.0			
	Control	Student	78	74.3			
	Control	Employed	27	25.7			
	Control	Unemployed	0	0.0			
Income Status	Patient	Very Low	34	34.0	68.181	4	<0.001
	Patient	Low	33	33.0			
	Patient	Moderate	19	19.0			
	Patient	High	9	9.0			
	Patient	Very High	5	5.0			
	Control	Very Low	4	3.8			
	Control	Low	17	16.2			
	Control	Moderate	14	13.3			
	Control	High	29	27.6			
	Control	Very High	41	39.0			

χ^2^, Chi-Square statistic. Groups differed significantly in educational attainment, profession, and income level (all p < 0.001).

Among AV patients, GAGS severity was mild in 20 (20.0%), moderate in 54 (54.0%), and severe in 26 (26.0%) patients. Mean disease duration was 3.69 ± 3.94 years. Regarding prior treatment, 43.0% were treatment-naïve, 20.0% had received topical therapy alone, 18.0% had received systemic antibiotics combined with topical agents, and 19.0% had used systemic isotretinoin.

### Comparison of scale scores between groups

AV patients demonstrated markedly and significantly higher DERS-16 scores compared to healthy controls (M = 39.55, SD = 15.33 vs. M = 15.18, SD = 11.27; t(203) = 13.01, p < 0.001; Cohen’s *d* = 1.818), representing a large effect. Total EDE-Q-13 scores were significantly lower in the patient group (M = 4.33, SD = 3.52 vs. M = 10.26, SD = 9.16; t(203) = −6.054, p < 0.001; Cohen’s *d* = −0.846). Critically, EDE-Q-13-BED subscale scores were significantly higher in AV patients than in controls (M = 1.47, SD = 1.66 vs. M = 0.92, SD = 1.06; t(203) = 2.849, p = 0.005; Cohen’s *d* = 0.398), a dissociation pointing to a qualitatively distinct eating pathology profile in AV (Table 3).

**Table 3:** Comparison of DERS-16, EDE-Q-13, and EDE-Q-13-BED scores between patient and control groups.

Scale	Group	Mean (SD)	*t*	df	p	Cohen’s *d*
DERS-16	Patient	39.55 (15.33)	13.01	203	<0.001	1.818
	Control	15.18 (11.27)				
EDE-Q-13 (Total)	Patient	4.33 (3.52)	−6.054	203	<0.001	−0.846
	Control	10.26 (9.16)				
EDE-Q-13-BED	Patient	1.47 (1.66)	2.849	203	0.005	0.398
	Control	0.92 (1.06)				

DERS-16, Difficulties in Emotion Regulation Scale-Brief Form; EDE-Q-13, Eating Disorder Examination Questionnaire short form; EDE-Q-13-BED, BED subscale; M, Mean; SD, Standard Deviation.

### Within-patient correlations

Within the AV patient group, all pairwise scale correlations were statistically significant (all p < 0.01). AQLS demonstrated a moderate positive correlation with DERS-16 (*r* = 0.591) and EDE-Q-13-BED (*r* = 0.418). DERS-16 was moderately correlated with both EDE-Q-13 (*r* = 0.410) and EDE-Q-13-BED (*r* = 0.468). The strongest correlation was observed between EDE-Q-13 total and BED subscale scores (*r* = 0.626). Correlations are presented in Table 4.

**Table 4:** Pearson correlation matrix: AQLS, DERS-16, EDE-Q-13, and EDE-Q-13-BED in the acne vulgaris group (n = 100).

	AQLS	DERS-16	EDE-Q-13
DERS-16	*r* = 0.591^a^ (p < 0.01)	‒	
EDE-Q-13	*r* = 0.343^a^ (p < 0.01)	*r* = 0.410^a^ (p < 0.01)	‒
EDE-Q-13-BED	*r* = 0.418^a^ (p < 0.01)	*r* = 0.468^a^ (p < 0.01)	*r* = 0.626^a^ (p < 0.01)

a All correlations significant at p < 0.01 (two-tailed). AQLS, Acne Quality of Life Scale.

### Multivariate regression analysis

For DERS-16, the overall model was significant (F(6, 198) = 28.330, p < 0.001, R²adj = 0.446). Group membership was the sole statistically significant predictor (β = 0.640, p < 0.001); no sociodemographic covariate reached significance (Table 5). This finding suggests that the elevated emotion dysregulation observed in AV patients represents a genuine disease-associated phenomenon largely independent of sociodemographic composition.

**Table 5:** Multivariate linear regression analysis: predictors of DERS-16 scores (n = 205).

Variable	B	SE	β	*t*	p	95% CI
Age	0.30	0.27	0.072	1.12	0.265	[−0.23, 0.82]
Education	−0.01	1.19	−0.000	−0.004	0.997	[−2.36, 2.35]
Occupation	−1.75	1.79	−0.058	−0.977	0.331	[−5.29, 1.79]
Income	−0.87	0.85	−0.069	−1.03	0.303	[−2.53, 0.79]
BMI	−0.11	0.27	−0.027	−0.41	0.682	[−0.65, 0.43]
Group^a^ (Patient = 1)	23.96^a^	2.47^a^	0.640^a^	9.70^a^	<0.001^a^	[19.12, 28.80]^a^

a R²adj = 0.446, F(6, 198) = 28.330, p < 0.001. Bold, Statistically significant predictor. B, Unstandardized coefficient; SE, Standard Error; β, Standardized Coefficient; CI, Confidence Interval.

For total EDE-Q-13, the model was significant (F(6, 198) = 8.637, p < 0.001, R²adj = 0.183). Two independent predictors emerged: group membership (β = −0.374, p < 0.001) and BMI (β = 0.196, p = 0.006; Table 6). The negative beta for group membership confirms that the unadjusted between-group EDE-Q-13 difference is substantially confounded by the socioeconomic and educational advantages of the control group.

**Table 6:** Multivariate linear regression analysis: predictors of EDE-Q-13 scores (n = 205).

Variable	B	SE	β	*t*	p	95% CI
Age	0.04	0.14	0.020	0.28	0.778	[−0.23, 0.30]
Education	0.79	0.61	0.103	1.30	0.194	[−0.41, 1.99]
Occupation	0.25	0.92	0.018	0.28	0.781	[−1.55, 2.05]
Income	−0.54	0.43	−0.092	−1.26	0.207	[−1.39, 0.30]
BMI^a^	0.39^a^	0.14^a^	0.196^a^	2.76^a^	0.006^a^	[0.11, 0.66]^a^
Group^a^ (Patient = 1)	−6.47^a^	1.25^a^	−0.374^a^	−5.17^a^	<0.001^a^	[−8.93, −4.01]^a^

a R²adj = 0.183, F(6, 198) = 8.637, p < 0.001. Bold, Statistically significant predictors.

To further characterize the independence of the binge-eating phenotype from sociodemographic confounders, a third multivariate linear regression model was constructed with EDE-Q-13-BED subscale score as the dependent variable, with group membership as the primary independent variable, and age, educational attainment, occupational status, household income, and BMI entered simultaneously as covariates. The overall model was statistically significant (F(6, 198) = 4.456, p < 0.001, R²adj = 0.092). Critically, even after controlling for all five sociodemographic and anthropometric covariates, group membership remained an independent and statistically significant predictor of EDE-Q-13-BED scores (B = 0.49, β = 0.175, p = 0.045, 95% CI for B [0.01, 0.98]), with the positive coefficient indicating that AV patients exhibited meaningfully higher binge-eating subscale scores than controls. BMI also emerged as a significant independent predictor (B = 0.09, β = 0.227, p = 0.002, 95% CI for B [0.03, 0.14]), consistent with its role identified in the EDE-Q-13 total score model. No other sociodemographic variable contributed independently. These findings confirm that the elevated binge-eating pathology observed in AV patients is not attributable to the sociodemographic composition of the groups but represents a disease-associated phenotype independent of educational attainment, occupational status, income level, age, and BMI (Table 7).

**Table 7:** Multivariate linear regression analysis: predictors of EDE-Q-13-BED scores (n = 205).

Variable	B	SE	β	*t*	p	95% CI
Age	0.02	0.03	0.057	0.60	0.547	[−0.04, 0.07]
Education	−0.17	0.12	−0.121	−1.41	0.161	[−0.40, 0.07]
Occupation	0.06	0.18	0.030	0.32	0.747	[−0.30, 0.41]
Income	0.02	0.08	0.020	0.23	0.821	[−0.15, 0.18]
BMI	**0.09**	**0.03**	**0.227**	**3.13**	**0.002**	**[0.03, 0.14]**
Group (Patient = 1)	**0.49**	**0.25**	**0.175**	**2.02**	**0.045**	**[0.01, 0.98]**

R²adj = 0.092, F(6, 198) = 4.456, p < 0.001. Bold, Statistically significant predictors; B, Unstandardized coefficient; SE, Standard Error; β, Standardized coefficient; CI, Confidence Interval. BMI, Body Mass Index.

### Ancillary within-patient findings

Two ancillary subgroup observations within the patient group provide additional contextual support for the principal analyses. Patients who had previously received systemic isotretinoin (n = 19) reported significantly greater subjective QoL impairment than all other treatment subgroups combined (AQLS: M = 25.21, SD = 8.52 vs. M = 19.95, SD = 8.82; t(98) = 2.335, p = 0.021), with no corresponding differences observed on DERS-16, EDE-Q-13, or EDE-Q-13-BED (all p > 0.05). This pattern is consistent with the interpretation that elevated QoL impairment does not mechanistically require a proportionate psychological response in the absence of concomitant emotion dysregulation ‒ and that the QoL-dysregulation linkage operates probabilistically rather than deterministically. Additionally, BMI category was a significant independent predictor of eating psychopathology within the patient group: patients with BMI ≥ 20 kg/m^2^ demonstrated significantly higher EDE-Q-13 (F(1, 98) = 9.358, p < 0.001, η²p = 0.162) and EDE-Q-13-BED scores (F(1, 98) = 4.446, p = 0.014, η²p = 0.084) relative to patients with BMI < 20 kg/m^2^, a finding concordant with the independent role of BMI identified in the multivariate regression models. These ancillary observations neither alter the principal conclusions of the study nor confound the mediation analyses presented below, in which GAGS ‒ not BMI ‒ serves as the designated independent variable.

### Mediation analysis

Two bootstrapped mediation analyses were conducted within the AV patient group (n = 100) using the Hayes PROCESS framework (Model 4) with 5,000 resamples,^12^ with GAGS as the independent variable, AQLS as the hypothesized mediator, and DERS-16 (Model 1) and EDE-Q-13 (Model 2) as respective outcomes. To ensure rigorous control for potential socioeconomic confounders within the patient sample, all three sociodemographic variables that showed statistically significant between-group differences ‒ educational attainment, occupational status, and household income ‒ were incorporated into both PROCESS models as covariates. Model parameters, path coefficients (a, b, c, and c’ paths), and bias-corrected bootstrapped 95% confidence intervals are presented in Table 8.

**Table 8:** Bootstrapped mediation analysis results: potential mediating role of AQLS (Hayes PROCESS Model 4, k = 5,000 resamples; adjusted for educational attainment, occupational status, and income level).

Path	β	SE	*t*	p	95% Bootstrap CI	Result
	*Model 1: GAGS → AQLS → DERS-16 (adjusted for education, occupation, income)*					
GAGS → AQLS (a path)	2.663	1.286	2.07	0.041	‒	
AQLS → DERS-16 (b path)	1.031	0.144	7.18	<0.001	‒	
GAGS → DERS-16 (total, c)	1.977	2.228	0.89	0.377	‒	
GAGS → DERS-16 (direct, c')	−0.770	1.840	−0.42	0.677	‒	Non-significant
**Indirect effect (AQLS mediates)**	**2.747**	‒	‒	‒	**[0.164, 5.837]**	**Significant**
	***Model 2: GAGS → AQLS → EDE-Q-13 (adjusted for education, occupation, income)***					
GAGS → AQLS (a path)	2.663	1.286	2.07	0.041	‒	
AQLS → EDE-Q-13 (b path)	0.126	0.038	3.28	0.001	‒	
GAGS → EDE-Q-13 (total, c)	0.670	0.506	1.32	0.188	‒	
GAGS → EDE-Q-13 (direct, c')	0.334	0.493	0.68	0.499	‒	Non-significant
**Indirect effect (AQLS mediates)**	**0.336**	‒	‒	‒	**[0.036, 0.836]**	**Significant**

Bootstrap CIs are bias-corrected 95% CIs. Indirect effect is significant when CI excludes zero. All models adjusted for educational attainment, occupational status, and household income as covariates. GAGS, Global Acne Grading System; AQLS, Acne Quality of Life Scale; DERS-16, Difficulties in Emotion Regulation Scale-Brief Form; EDE-Q-13, Eating Disorder Examination Questionnaire short form.

In Model 1 (GAGS → AQLS → DERS-16), GAGS significantly predicted AQLS (β = 2.663, *t* = 2.07, p = 0.041), and AQLS in turn significantly predicted DERS-16 (β = 1.031, *t* = 7.18, p < 0.001). Neither the total (β = 1.977, p = 0.377) nor the direct (β = −0.770, p = 0.677) effect of GAGS on DERS-16 was statistically significant. The bootstrapped indirect effect through AQLS was significant and robust (β = 2.747, 95% CI [0.164, 5.837]), indicating that subjective QoL impairment may be associated with the relationship between objective acne severity and emotion dysregulation, even after adjustment for educational attainment, occupational status, and income.

In Model 2 (GAGS → AQLS → EDE-Q-13), GAGS significantly predicted AQLS (β = 2.663, p = 0.041), and AQLS significantly predicted EDE-Q-13 (β = 0.126, p = 0.001). Total (β = 0.670, p = 0.188) and direct (β = 0.334, p = 0.499) effects of GAGS on EDE-Q-13 were non-significant, while the indirect effect through AQLS was significant and retained its magnitude after covariate adjustment (β = 0.336, 95% CI [0.036, 0.836]). These findings are consistent with a pattern in which the association between objective acne severity and eating psychopathology is primarily transmitted through the patient’s subjective QoL experience, rather than through a direct pathway.

## Discussion

This study examined the associations between objective acne severity, subjective QoL impairment, emotion dysregulation, and binge-eating pathology in a cross-sectional sample of AV patients and matched controls. The principal observations ‒ markedly elevated DER and EDE-Q-13-BED scores in AV patients, the absence of a statistically significant direct association between objective severity and psychological outcomes, and the significant indirect associations of subjective QoL in both relationships ‒ collectively suggest that reliance on lesion burden alone may be insufficient for estimating psychological risk in clinical practice.

### The sociodemographic context: why did controls score higher on general eating disorder psychopathology?

The most immediately counterintuitive finding of the present study is that total EDE-Q-13 scores were significantly lower in AV patients than in healthy controls. This observation requires contextualization before any clinical inference can be drawn. The control group was characterized by substantially higher educational attainment and household income than the patient group ‒ a sociodemographic imbalance that, while systematically addressed in the multivariate regression models, exerts a well-documented independent influence on restraint-based eating psychopathology. Higher socioeconomic status in young populations ‒ particularly among women ‒ is independently associated with greater exposure to and internalization of the sociocultural thin ideal, heightened weight-related preoccupation, and elevated prevalence of cognitive dietary restraint. The EDE-Q-13 total score, as an instrument whose primary sensitivity lies in capturing dietary restraint, weight concern, and shape concern, is accordingly susceptible to this restraint-driven phenotype and may reflect sociocultural pressures as much as genuine clinical psychopathology in comparisons across socioeconomically disparate groups.

This interpretation is consistent with the multivariate regression findings, in which group membership independently predicted lower total EDE-Q-13 scores in the patient group after full adjustment for educational attainment, occupational status, household income, age, and BMI (β = −0.374, p < 0.001), and in which BMI emerged as an additional independent predictor (β = 0.196, p = 0.006). The sociodemographic imbalance between groups should therefore be understood not as a confound that undermines the principal findings, but as a contextual factor that ultimately clarifies them. Despite belonging to a socioeconomic group less exposed to thin-ideal sociocultural pressures, AV patients still exhibited a specific and clinically meaningful eating pathology profile ‒ one characterized by elevated binge-eating behavior rather than dietary restraint. It is precisely this dissociation that constitutes the study’s most clinically relevant contribution to the dermatological literature.

### A distinct eating pathology profile in acne: contextualization within the prior literature

The co-elevation of DERS-16 and EDE-Q-13-BED scores in AV patients, occurring alongside a paradoxical reduction in restraint-based eating psychopathology on the total EDE-Q-13, converges with, extends, and in important respects refines the findings of prior investigations in this area. Considered together, the existing literature reveals a consistent ‒ though not uniformly operationalized ‒ signal linking acne-related psychological distress to disordered eating behavior, and the present study offers a methodological framework capable of disambiguating the specific phenotype involved.

Oner and Hacinecipoglu^13^ provided an early and influential demonstration that AV patients face elevated eating disorder risk relative to healthy controls, reporting higher EAT-40 scores in a sample of 232 patients compared to 314 controls. Critically, their findings anticipated a key observation of the present study: eating disorder risk in their cohort was associated with QoL impairment as measured by the AQLS, but not with objective acne severity as measured by the GAGS ‒ a pattern directly replicated and formally extended in the present bootstrapped mediation analyses. However, the EAT-40, as a global screening instrument, does not permit disaggregation of restraint-based from affect-driven binge-eating phenotypes, leaving the specific nature of the disordered eating profile in AV patients uncharacterized. Kaya et al.^6^ similarly observed significant elevations in restraint eating and shape concern among 100 AV patients relative to 86 controls using the EDE-Q, identifying OCD symptoms and depression as key psychiatric correlates. While this study advanced understanding of the psychiatric comorbidity landscape in AV, its primary finding of elevated restraint-based pathology may partly reflect the sociocultural and sociodemographic characteristics of the comparison sample rather than a disease-specific eating phenotype ‒ a limitation that the present study’s disaggregated analytical approach was specifically designed to address. Karaagac et al.^7^ reached a convergent conclusion from a different analytical vantage point, finding no direct association between GAGS severity and eating attitudes in a cross-sectional sample of 81 AV patients assessed with the TFEQ-21, while identifying depression and anxiety as significant correlates of emotional and uncontrolled eating subscales ‒ a pattern thematically consistent with the affect-driven, emotion dysregulation-mediated pathway proposed in the present study.

The role of emotion dysregulation itself as a psychodermatological variable in AV was most directly examined by Cengiz and Gurel,^3^ who administered the DERS-16 and AQLS to 141 AV patients and 102 controls and found that DER was associated with QoL impairment more strongly than clinical severity, with no significant GAGS-DER correlation identified ‒ a finding directly replicated in the present study and now extended through formal mediation analysis to include binge-eating pathology as a downstream psychological outcome. An apparent divergence in the literature warrants specific discussion, however. Turan et al.^5^ reported that GAGS scores were positively correlated with total DERS scores and with psychopathology symptom burden in a sample of 96 adolescents with AV, a finding that stands in contrast to the null GAGS–DERS-16 direct association observed in the present study. Several factors may account for this discrepancy. First, the Turan et al.^5^ sample was exclusively adolescent in composition, a developmental period in which acne lesion visibility may carry heightened interpersonal and social-evaluative salience ‒ potentially rendering the objective-subjective severity dissociation less pronounced than in a mixed-age sample spanning adolescence and adulthood, as in the present study. Second, Turan et al.^5^ employed the full 36-item DERS rather than the 16-item brief form, and the two instruments, while psychometrically related, are not equivalent in their subscale structure or their sensitivity to specific regulatory dimensions; differential instrument sensitivity may therefore contribute to divergent findings across studies. Third, the specific clinical and healthcare context of the Turan et al.^5^ sample ‒ a pediatric setting with a predominantly treatment-seeking adolescent population ‒ may have selected for patients with more severe or distressing disease, potentially attenuating the otherwise typical dissociation between objective lesion burden and subjective psychological impact. Taken together, these methodological and developmental contextual differences suggest that the GAGS-DER association may be moderated by age and developmental stage, with the QoL-mediated pathway identified in the present study more characteristic of a mixed-age or predominantly young-adult outpatient population.

### Subjective QoL as the key clinical target

The observation that GAGS severity showed no statistically significant direct association with psychological outcomes ‒ and that AQLS was associated with significant indirect effects in both the GAGS–DERS-16 and GAGS–EDE-Q-13 relationships ‒ has potentially substantive implications for how dermatologists assess psychological risk in routine clinical practice. A high GAGS score in a patient with preserved QoL may not predict significant psychological morbidity; conversely, a moderate GAGS score in a patient with severely impaired QoL may be associated with meaningful emotion dysregulation and binge-eating risk. Rather than implying a deterministic or invariant pathway, these findings suggest a potential indirect pathway through which objective lesion burden may come to be associated with psychological outcomes ‒ one in which the patient’s subjective appraisal of disease-related QoL impairment appears to be the more proximal and clinically actionable variable.

These findings align with and extend the work of Cengiz and Gurel,^3^ who similarly found DER to be a stronger correlate of QoL than objective severity, and with Oner and Hacinecipoglu,^13^ who observed that eating disorder risk was associated with QoL impairment rather than GAGS scores. The present study extends these contributions by formally evaluating the indirect role of AQLS through bootstrapped mediation analysis, providing a more methodologically rigorous characterization of the association structure. Notably, the persistence of both the binge-eating phenotype and the mediation pathways after stringent simultaneous adjustment for all sociodemographic variables that differed significantly between groups ‒ educational attainment, occupational status, and household income ‒ strengthens the internal validity of these observations and reduces the plausibility that they are attributable to compositional differences between the groups. In clinical practice, these findings suggest that routine QoL assessment ‒ using a validated instrument such as the AQLS ‒ may serve as a more sensitive and efficient triage tool for identifying patients at psychological risk than objective severity grading alone. Patients reporting high AQLS impairment, regardless of GAGS score, may benefit from targeted psychological screening encompassing DER and BED alongside the established evaluation of depression and anxiety.

The subgroup observation that patients with objectively severe acne but paradoxically low AQLS scores demonstrated significantly lower DERS-16 scores (M = 28.86, SD = 11.09 vs. M = 46.42, SD = 18.04; *t* = −2.395, p = 0.025) further supports this interpretation, suggesting that preserved subjective QoL may be associated with attenuated emotion dysregulation even in the context of high lesion burden ‒ and reinforcing the view that subjective disease appraisal, rather than objective lesion severity, may be the more proximal correlate of psychological outcomes in AV.

### A distinct eating pathology profile in acne: clinical implications for dietary counselling

The co-elevation of DERS-16 and EDE-Q-13-BED scores in AV patients, occurring alongside a paradoxical reduction in restraint-based eating psychopathology, may represent the study’s most directly actionable contribution for practicing dermatologists. Restraint-based eating disorders are characteristically associated with thin-ideal internalization, body dissatisfaction, and the cognitive motivation to restrict dietary intake. Binge-eating disorder, by contrast, is typically associated with negative affect and the use of eating as a behavioral strategy for the short-term relief of emotional distress,^4^ and the present findings are consistent with this affect-driven model operating within the AV population.

This distinction carries a direct clinical implication for dermatological practice: clinicians who provide dietary guidance to AV patients ‒ particularly low-glycaemic-index or dairy-free regimens ‒ should consider the possibility that a subset of patients may present features of DER and affect-driven binge eating. In such individuals, restrictive dietary prescriptions may be associated with compounding of existing affective dysregulation and may potentially reinforce binge-eating behavior rather than ameliorate it. A brief inquiry about the patient’s relationship with food and emotional state, or the systematic use of a validated screening tool such as the EDE-Q-13, may help identify this subgroup and inform a more tailored, multidisciplinary management approach.

### Study limitations

Several limitations of the present study warrant explicit acknowledgement. The cross-sectional design is the most fundamental methodological constraint: it precludes the establishment of causal directionality and, critically, does not permit the determination of temporal ordering among the variables examined. While the bootstrapped mediation analyses are statistically consistent with the proposed indirect pathway from objective severity through subjective QoL to psychological outcomes, they cannot confirm that this sequence operates in the direction specified, nor that it is causal in nature. Furthermore, alternative and bidirectional models cannot be excluded; for instance, emotion dysregulation may itself influence subjective QoL appraisal, or unmeasured psychological vulnerabilities might independently shape both skin-related distress and affect-driven eating patterns. Longitudinal designs incorporating repeated assessment of all variables would be required to formally adjudicate among these competing models and establish temporal precedence.

All psychological outcomes were assessed via self-report questionnaires, which are susceptible to response bias and social desirability effects; no structured clinical diagnostic interviews were conducted to confirm formal psychiatric diagnoses, and the presence of subthreshold or undiagnosed psychiatric comorbidity in either group cannot be excluded. The most significant sociodemographic limitation is the substantial imbalance between groups in educational attainment, occupational status, and household income. Multivariate regression models explicitly controlled for all identified confounders, and principal findings remained robust after adjustment; however, residual confounding from unmeasured sociodemographic variables ‒ including cultural attitudes toward body image, dietary behavior, and help-seeking ‒ cannot be excluded. This imbalance should be understood as reflecting the sociodemographic composition of the dermatology outpatient population in this setting, and as a contextual factor that ultimately clarifies rather than invalidates the study’s conclusions. The predominantly female composition (80.0%) limits generalizability to male AV populations, and the single-center design restricts the external validity of findings to other clinical settings and geographical contexts.

## Conclusion

This study provides evidence that the psychological burden of acne vulgaris may involve a specific profile characterized by the co-elevation of emotion dysregulation and binge-eating pathology ‒ a profile that appears qualitatively distinct from the restraint-based eating disorders more typically associated with sociocultural pressures in higher socioeconomic status populations. Crucially, objective acne severity was not directly associated with psychological outcomes in this sample; rather, subjective QoL impairment was associated with significant indirect effects on both the acne severity-emotion dysregulation and acne severity-eating pathology relationships, suggesting a potential indirect pathway through which lesion burden may come to be associated with psychological morbidity. Importantly, these indirect effects remained statistically significant and retained their magnitude after stringent simultaneous adjustment for socioeconomic variables, lending additional support to the robustness of the observed associations within the patient population. Given the cross-sectional design, causal attribution is not warranted, and bidirectional or alternative associative models cannot be excluded on the basis of the present data alone.

These findings highlight the potential clinical utility of routinely assessing patient-reported QoL in AV ‒ using validated instruments such as the AQLS ‒ as a more sensitive indicator of psychological risk than lesion grading alone. Dermatologists providing dietary counselling to AV patients should consider whether patients present features of emotion dysregulation or binge-eating pathology, as restrictive dietary advice may be counterproductive in this subgroup and its use should be informed by a broader assessment of the patient’s psychological profile. The comprehensive management of acne vulgaris may, on the basis of these findings, benefit from a multidisciplinary approach that integrates validated QoL and psychological screening into routine care, irrespective of clinical lesion burden.

## ORCID ID

Cagdas Boyvadoglu: 0000-0003-2087-2861

Cagla Boyvadoglu: 0000-0003-0586-5399

Ayse Serap Karadag: 0000-0003-4333-8274

## Authors' contributions

Cagdas Boyvadoglu: Data collection, analysis and interpretation; manuscript critical review; preparation and writing of the manuscript; study conception and planning.

Cagla Boyvadoglu: Manuscript critical review; study conception and planning.

Ayse Serap Karadag: Effective participation in research orientation; manuscript critical review.

## Ethical approval

This study was conducted in accordance with the principles of the Declaration of Helsinki. Ethical approval was granted by the Scientific Research Ethics Committee of Adana City Training and Research Hospital (Approval Code: 310, 2 January 2025, for the patient group; Approval Code: 667, 21 August 2025, for the control group). Written informed consent was obtained from all participants prior to enrolment.

## Data availability

The datasets generated and/or analyzed during the current study are available from the corresponding author on reasonable request.

## Financial support

None declared.

## Research data availability

The data are available from the corresponding author on reasonable request.

## Declaration of generative AI and AI-assisted technologies in the manuscript preparation process

During the preparation of this work, the authors used Large Language Model (LLM)-based AI-assisted tools solely for the purpose of English language editing, proofreading, and improving the readability and flow of the manuscript. These tools were not used for data analysis, data interpretation, or generation of any scientific content. After using these tools, the authors reviewed and edited all content as needed and take full responsibility for the integrity and accuracy of the published article.

## Conflicts of interest

None declared.
